# Data-Driven Identification of Brain–Behavioral and Sociodemographic Predictors of Anxiety Severity in Children Using Machine Learning

**DOI:** 10.1016/j.jaacop.2025.05.005

**Published:** 2025-06-25

**Authors:** Ann M. Iturra-Mena, Melanie Wall, Sherry Y.H. Chen, Jason S. Moser, Maria Muzik, Katherine Rosenblum, Kate D. Fitzgerald

**Affiliations:** a Columbia University, New York, New York; b Michigan State University, East Lansing, Michigan; c University of Michigan, Ann Arbor, Michigan; d New York State Psychiatric Institute, New York, New York

**Keywords:** anxiety prediction, EEG biomarkers, cognitive control, machine learning, child psychiatry

## Abstract

**Objective:**

Childhood anxiety is highly prevalent and linked to cognitive control deficits and sociodemographic risk factors. However, how these factors contribute and interact remain unclear. This study used a data-driven machine learning approach to identify the strongest neural, behavioral, and sociodemographic predictors of childhood anxiety severity.

**Method:**

A sample of 181 children (aged 4-10 years) was drawn from 3 studies examining anxiety and error monitoring. Anxiety severity was assessed using the Child Behavior Checklist *DSM*-Oriented Anxiety Scale. Predictors included cognitive control markers derived from electroencephalogram recordings and behavioral measures, both obtained during a Go/NoGo task, along with sociodemographic risk factors. Random Forest, Support Vector Regression, and XGBoost models were compared using cross-validation. Partial dependence plots were used to examine predictor–outcome relationships, and interactions were analyzed using H-statistic.

**Results:**

Random Forest yielded optimal prediction accuracy. Error positivity (Pe) emerged as the strongest predictor, followed by single-mother status, frontal theta power, post-error accuracy, and occipital post-error alpha power. Single-mother status strengthened the relationship between post-error alpha power and anxiety, indicating that impaired post-error attentional mechanisms may be more pronounced in anxious children from single-mother households. Low frontal theta power amplified the Pe–anxiety relationship, suggesting that reduced inhibitory control may intensify anxiety-related error awareness.

**Conclusion:**

A specific interaction among neural indices of cognitive control (Pe, frontal theta, occipital post-error alpha), behavioral performance (post-error accuracy), and a sociodemographic factor (single/unpartnered mother) predicted childhood anxiety. These findings highlight the importance of considering both neural mechanisms and environmental context in guiding diagnosis and treatment, offering insights beyond traditional hypothesis-driven approaches.

**Diversity & Inclusion Statement:**

We worked to ensure sex and gender balance in the recruitment of human participants. We worked to ensure race, ethnic, and/or other types of diversity in the recruitment of human participants. We worked to ensure that the study questionnaires were prepared in an inclusive way. One or more of the authors of this paper self-identifies as a member of one or more historically underrepresented racial and/or ethnic groups in science. One or more of the authors of this paper self-identifies as living with a disability. One or more of the authors of this paper self-identifies as a member of one or more historically underrepresented sexual and/or gender groups in science. One or more of the authors of this paper received support from a program designed to increase minority representation in science.

## Plain language summary

This study used brain and behavioral data from 181 children ages 4 to 10 to identify which factors best predict anxiety symptoms. Using machine learning, researchers found that increased brain response to mistakes, lower attention-related brain activity, and living in a single-mother household were the strongest predictors. These findings highlight the importance of considering both brain function and family context when identifying children at risk for anxiety.

Clinically significant anxiety represents the most commonly occurring mental health challenge during early development,^1^ with nearly one-third of children meeting criteria for an anxiety disorder by their teenage years.^2^ Childhood anxiety often persists into adulthood, leading to poorer mental and physical health, lower educational and employment success, and increased societal economic costs.^3^ This underscores the need for quantifiable, mechanistically based markers that can guide early detection, intervention, and prevention strategies. To guide the identification of such markers, the Research Domain Criteria (RDoC) framework suggests linking symptoms such as anxiety to underlying brain circuits associated with psychologically relevant constructs,^4^ such as cognitive control—that is, the ability to regulate thoughts and actions in accordance with internally represented behavioral goals, especially in the face of competing demands, distractions, or habitual responses.^5^

Abnormalities in cognitive control processes have been implicated in early emerging anxiety.^6^^,^^7^ However, studies examining brain–behavioral markers of cognitive control alongside sociodemographic risk to predict subclinical-to-clinical levels of childhood anxiety are needed, as they could provide a multilevel understanding of the mechanisms underlying early-onset symptoms. Existing evidence indicates that growing up in poverty has a negative impact on cognitive control skills in children^8^ and increases risk for early-onset psychopathology, including anxiety.^9^ Age and gender also have impacts on the presentation of anxiety symptoms and the development of cognitive function.^10^^,^^11^ In addition, single-parent status and maternal psychopathology (eg, depression) are strong risk factors for internalizing disorders in children and adolescents.^12^, ^13^, ^14^, ^15^

Electroencephalography (EEG) offers complementary markers of cognitive control, spanning error processing, inhibitory control, and time–frequency dynamics, which may predict anxiety risk. Error processing is captured by 2 key event-related potentials: the Error-Related Negativity (ERN) and Error Positivity (Pe). The ERN, occurring 100 milliseconds post error in the dorsal anterior cingulate cortex (dACC), reflects neural mechanisms underlying post-error attention.^16^, ^17^, ^18^ This component shows age-dependent anxiety associations, with increased amplitude relating to greater symptom severity in adolescents but decreased amplitude in younger children.^10^^,^^19^ The Pe, appearing 200 to 500 milliseconds post error in centroparietal regions, reflects error awareness,^20^ with mixed evidence for its relationship with childhood anxiety.^21^^,^^22^ The N200 (or N2) indexes conflict monitoring, with its amplitude reflecting the attentional control needed to resolve conflict and to inhibit incorrect responses, whereas the P300 (or P3) indicates response inhibition. Both components show alterations in anxious children, suggesting attention biases and hypervigilance.^23^ Time–frequency analyses provide additional insights through theta (4-7 Hz) and alpha (8-14 Hz) band activity. Midline frontal theta typically increases following errors, optimizing behavioral adjustments.^24^ Recent studies show that anxious children exhibit reduced theta activity and altered alpha patterns, including attenuated error-related alpha suppression^22^ and reduced alpha coherence,^25^ suggesting difficulties disengaging from internal worries. These neural patterns manifest behaviorally as excessive post-error slowing and reduced accuracy despite longer response times,^22^^,^^26^ aligning with theories that anxiety disrupts efficient cognitive control by impairing attentional mechanisms.^27^

To effectively integrate diverse brain–behavioral indices of cognitive control with anxiety-relevant sociodemographic variables for predicting anxiety severity, it is crucial to use statistical methods that are flexible, data driven, and capable of handling complex interactions. Traditional statistical approaches often impose constraints, such as limiting the number of predictors and interactions that can be tested simultaneously, assuming linear relationships, and requiring researchers to predefine relationships between variables and outcomes. In contrast, machine learning overcomes these limitations by capturing both linear and nonlinear relationships, modeling complex interactions among multiple predictors, and identifying the relative importance of each predictor in a data-driven manner. This approach aligns well with the RDoC framework, which emphasizes a dimensional and mechanistic understanding of anxiety severity by integrating multimodal data without relying on predefined diagnostic categories.

In this study, we used a data-driven approach with machine learning to identify patterns among multiple predictors. By integrating measures across multiple levels of analysis, as outlined in the RDoC framework, we aimed to model childhood anxiety severity across a subclinical-to-clinical range in a primarily community-based sample. This data-driven strategy addressed 3 key questions: (1) Which markers among neural, behavioral and sociodemographic measures emerge as the strongest predictors of anxiety severity when allowed to compete in importance? (2) What patterns of relationships between these predictors and anxiety severity are revealed when examined without *a priori* assumptions about their functional form? (3) What previously unidentified interactions among predictors emerge?

To address these questions, we leveraged machine learning’s ability to identify patterns in multidimensional data. This approach allowed us to determine the relative importance of predictors across a subclinical-to-clinical range of anxiety, to examine their relationships through partial dependence plots, and to quantify interactions using H-statistic analysis.

## Method

### Participants

In this cross-sectional study, we conducted secondary data analysis from a sample that included 181 children (56.9% female, aged 4-10 years) who participated in 3 consecutive studies by the same site and team examining error monitoring and anxiety.^10^^,^^28^^,^^29^ Each study used identical EEG paradigms, behavioral tasks, and clinical assessments. Quality control procedures for data integration ensured measurement consistency across studies, including identical clinical measures, EEG recording parameters, Go/NoGo task administration, and data processing pipelines. Eligibility required participants to have no history of head injuries, significant medical illnesses that could potentially confound study results or interfere with participation, neurodevelopmental delays, or medications that affect central nervous system functioning. Externalizing symptoms were permitted if anxiety was the primary source of distress. In the 3 studies, participants were recruited through a stratified sampling approach based on *DSM*-Oriented Anxiety Scale *T* scores from the Child Behavior Checklist (CBCL). Recruitment targeted participants with *T* scores >60 (clinically relevant), recruited primarily from the University of Michigan Child and Adolescent Psychiatry Clinic and Infant and Early Childhood Clinic, and with *T* scores <60 to ensure the inclusion of participants in the normative to subclinical range of anxiety, who were recruited primarily from the community. In one of the studies (N = 69), participants were also assessed using structured clinical interviews (K-SADS-PL). However, these data were not included in the present study, as they were not available for the entire sample (in this subsample, the correlation between the CBCL Anxiety scale and Kiddie Schedule for Affective Disorders and Schizophrenia–Present and Lifetime Version [K-SADS-PL] anxiety problems was *r* = 0.624, *p* = .001). The Institutional Review Board of the University of Michigan Medical School approved these studies.

#### Sample Size Estimation

Although traditional power calculations designed for inferential statistics are not directly applicable to machine learning models, we conducted an initial sample size estimation using G∗Power 3.1.^30^ Based on a multiple regression model with 26 predictors, detecting a medium effect size (f^2^ = 0.15) at α = 0.05 and power = 0.80 required a minimum of 175 participants. This estimation served as a conservative baseline for our machine learning approach including 181 subjects and 26 predictors.

### Procedures and Data Modalities

The primary outcome variable, child anxiety, was measured by mother report on the Child Behavior Checklist *DSM*-oriented Anxiety Problems subscale (CBCL-Anxiety *T* scores).^31^ The set of predictors included 26 variables derived from task performance metrics on the Go/NoGo Zoo Task, neural signatures of cognitive control (ie, EEG-derived metrics), and sociodemographic factors. The procedures are explained in detail in Supplement 1 (available online) and summarized briefly below. All predictors included in the model are summarized in Table S1, available online.

#### Go/NoGo Zoo Task

This task was selected to assess cognitive control, a key executive function that has been implicated in anxiety disorders.^10^^,^^22^ The Zoo Task measures response inhibition and error monitoring processes, which are often altered in anxious individuals. The child-friendly design using animal stimuli ensures age-appropriate engagement while maintaining robust psychometric properties for assessing cognitive control.^32^ The task’s parameters create a prepotent response tendency that requires active inhibitory control, making it particularly sensitive to individual differences in cognitive control abilities that may relate to anxiety symptoms. Children completed a computerized Zoo Task,^32^ responding to animal images while inhibiting responses to orangutans across 320 trials. Task performance metrics included false alarm rates, miss rates, post-error slowing, and post-error accuracy (detailed procedures in Supplement 1, available online).

#### EEG Signatures of Cognitive Control

EEG data were recorded using 17 Ag/AgCI scalp electrodes and processed using standard procedures (detailed in Supplement 1, available online). Key neural measures included the ERN and Pe, the stimulus-locked N200 and P300 components, and time–frequency measures of theta and alpha power computed using complex Morlet wavelet transformation. Post-error frontal synchrony was computed using phase locking values between frontal electrode pairs.

Table S2 (available online) summarizes all EEG and Zoo Task performance metrics used in the machine learning models.

#### Sociodemographic Factors

In addition to age of the child (in months) and sex assigned at birth, demographic data collected via questionnaires was compiled to generate separate indicators of demographic risk based on previous literature.^33^^,^^34^ Indicators were a single or unpartnered mother, low maternal education, racial minority status, and low household income (less than $25,000). Young maternal age (<22 years) has also been considered relevant,^33^^,^^34^ but in our sample all mothers were older than 22 years, so we excluded this indicator. Each indicator was included in the models as a separate categorical variable (1 = risk, 0 = no risk).

#### Maternal Psychopathology

Maternal depression symptoms, measured by the Beck Depression Inventory (BDI),^35^ were examined as an indicator of maternal psychopathology. Although both maternal depression and anxiety have been associated with children’s psychopathology,^12^, ^13^, ^14^^,^^36^ maternal depression was selected because of data availability in the 3 original studies.

### Statistical Analysis

Missing data were imputed using missForest,^37^ a nonparametric method based on random forest (Supplement 2, available online).

To identify the optimal predictive model for anxiety severity, we compared 3 machine learning algorithms suitable for regression and relatively small sample sizes: Random Forest (RF), Support Vector Regression (SVR), and XGBoost. All models were evaluated using 5-fold cross-validation (CV) in the caret R package (version 6.0-93). In each iteration, the dataset was split into 5 equal-sized folds, with 4 folds used for training (80%) and 1 fold for testing (20%). Each fold served as the test set once, and final performance metrics were averaged across iterations. The RF model was constructed with 1,000 trees, with mtry automatically tuned (tuneLength = 5). SVR used a radial basis function kernel with hyperparameters optimized via grid search. XGBoost parameters were similarly tuned via grid search over learning rate, tree depth, and subsample ratio. Model performance was evaluated using the root mean square error (RMSE) and *R*^*2*^. Statistical significance of model differences was tested via bootstrap resampling (1,000 iterations), generating empirical distributions for 95% confidence intervals and pairwise comparisons. Variable importance was assessed through permutation-based importance measures.

The relationships between predictors and anxiety were examined using partial dependence plots (pdp package), whereas interactions among the most important predictors were assessed with the Friedman *H* statistic (iml package) and further explored through moderation analysis using linear regression for interpretability.

## Results

### Participant Characteristics

As shown in Table 1, participants (N = 181, 56.9% female) had an average age of 6.26 years (SD = 1.29; range = 3.75-9.67). The sample was predominantly White non-Latino (69%), with 11% African American non-Latino and 20% of other races or ethnicities, including Latino. Regarding other sociodemographic risk factors, 33% came from single-mother households, 2.8% had mothers with low educational attainment, and 9.4% reported low household income. Table S3 (available online) shows the distribution of sociodemographic risk factors by racial/ethnic groups. The mean CBCL-Anxiety *T* scores were 56.74 (SD = 9.46, range = 50-90, skewness = 1.43), with 31% of children showing clinically relevant anxiety symptoms (*T* scores >60) and 69% showing normative to subclinical levels (*T* scores 50-60).

**Table 1:** Participant Characteristics (Demographics, Risk Factors, and Child Behavior Checklist Scores) (N = 181)

Sex	
Female	103 (56.9%)
Male	78 (43.1%)
Age, mo, mean (SD; Min-Max)	73.13 (15.53; 45-116)
Race/ethnicity	
Asian or Pacific Islander	5 (2.8%)
Biracial	26 (14%)
Black or African American	20 (11%)
Hispanic or Latino	5 (2.8%)
Native American	0 (0%)
Other	1 (0.6%)
White non-Hispanic or Latino	124 (69%)
Other risk factors	
Single/unpartnered mother	60 (33.1%)
Low maternal education	5 (2.8%)
Low household income	17 (9.4%)
BDI score mother [mean (SD; Min-Max)]	7.82 (8.27; 0-37)
CBCL *T* scores	
CBCL DSM Anxiety Scale, mean (SD; Min-Max)	56.74 (9.46; 50-90)
CBCL *DSM* Anxiety Scale *T* scores <60 (normative to subclinical)	125 (69%)
CBCL *DSM* Anxiety Scale *T* scores >60 (clinical level)	56 (31%)
CBCL-Internalizing, mean (SD; Min-Max)	49.3 (12.1; 29-76)
CBCL-Externalizing, mean (SD; Min-Max)	48.5 (10.9; 28-79)

CBCL = Child Behavior Checklist; Max = maximum; Min = minimum.

### Model Selection and Performance

We compared 3 machine learning algorithms predicting CBCL-Anxiety *T* scores: RF, SVR, and XGBoost. The RF model demonstrated competitive performance (RMSE = 8.264, *R*^*2*^ = 0.286) compared to SVR (RMSE = 8.355, *R*^*2*^ = 0.285) and XGBoost (RMSE = 8.308, *R*^*2*^ = 0.269). Bootstrap resampling with 1,000 iterations confirmed that RF significantly outperformed both SVR and XGBoost (*p* < .001), whereas there was no statistically significant difference between SVR and XGBoost models (*p* = .888) (Table S4, available online). In addition, RF is preferable over SVR in this context of limited sample size, because of its in-built out-of-bag estimation for training and testing, which provides a more robust validation approach without requiring separate test sets that would further reduce our effective sample size.

To test model specificity, we conducted parallel analyses predicting CBCL Internalizing and Externalizing scores. Although the RF model showed comparable explained variance across these broader domains (internalizing: *R*^*2*^ = 0.287; externalizing: *R*^*2*^ = 0.289), prediction errors were notably higher (internalizing: RMSE = 10.435; externalizing: RMSE = 9.550), suggesting that our model achieves better prediction accuracy specifically for anxiety while also capturing variance shared with broader patterns of childhood psychopathology.

### Importance of Predictors

As shown in Table 2, the Pe emerged as the most important predictor (standardized importance = 100), followed closely by the mother being single/unpartnered (95.65). Frontal theta power during NoGo stimuli (79.41), post-error accuracy (73.15), and occipital post-error alpha power (60.63) completed the top 5 predictors. This ranking suggests that neural markers of error processing and cognitive control, combined with sociodemographic risk factors, are particularly important for predicting anxiety severity in children.

**Table 2:** Variable importance final model for predicting CBCL-Anxiety

Ranking	Variable	Standardized importance
1	Pe	100.00
2	Single mother	95.65
3	FCz.Theta.Stim.NoGo	79.41
4	Post.Error.Acc	73.15
5	Occipital.Post.Error.Alpha	60.63
6	fa.ER	59.72
7	fa.RT	55.55
8	PLV_F3.F4_Alpha_NoGo_Error	50.52
9	PLV_F3.F4_Theta_NoGo_Error	48.91
10	PLV_F4.FCz_Theta_NoGo_Error	48.88
11	BDI.score	46.68
12	PLV_F3.FCz_Alpha_NoGo_Error	40.98
13	Age.months	38.66
14	FCz.Post.Error.Theta	35.44
15	N200	35.14
16	hit.RT	32.80
17	PLV_F3.FCz_Theta_NoGo_Error	32.28
18	Post.Error.RT	31.29
19	PLV_F4.FCz_Alpha_NoGo_Error	30.06
20	ERN	29.79
21	miss.ER	29.63
22	P300	24.78
23	incomeRisk	6.07
24	Racerisk	2.56
25	Edurisk	1.47
26	Sex	0.00

Note: Variable importance scores for the Random Forest model were computed using permutation-based importance and standardized for easier interpretation. The most important variable (Pe) is set to 100, with all other features scaled relative to it. Higher values indicate greater importance in predicting CBCL-Anxiety T scores. CBCL = Child Behavior Checklist; other abbreviations in Table S1, available online.

### Predictor–Outcome Relationships

**Figure 1:**
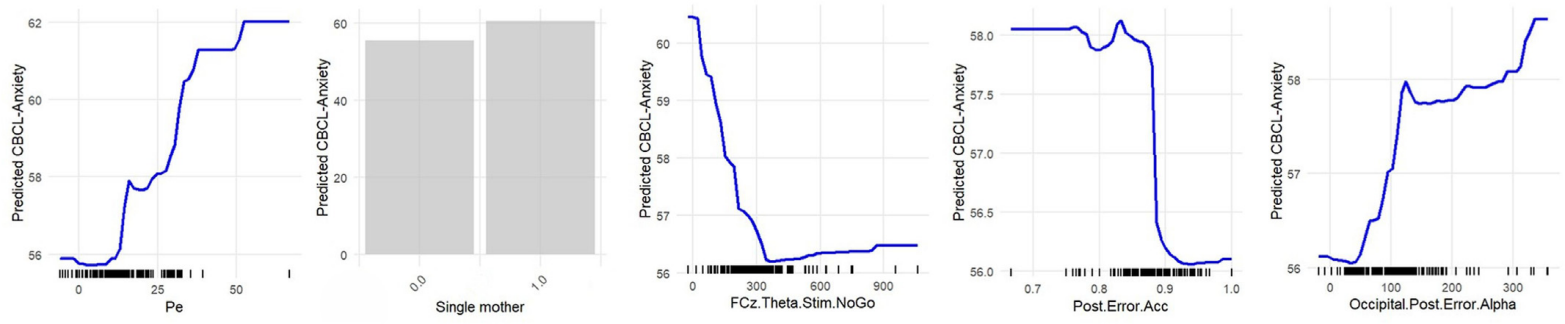
Partial Dependence Plots for the Most Important Predictors of CBCL-Anxiety ***Note:****These plots visually represent the relationship between the target variable and the 5 top predictors in the model with the best performance (Random Forest). The second most important predictor, single/unpartnered mother risk, is categorical and is therefore presented as a bar plot, showing the mean predicted values across categories (1 = single/unpartnered mother; 0 = partnered mother). CBCL = Child Behavior Checklist; FCz.Theta.Stim.NoGo = stimulus-locked (150-400 ms) theta (4-7 Hz) power for NoGo trials channel FCz; Occipital.Post.Error.Alpha = response-locked (200-500 ms) average alpha (8-14 Hz) power for NoGo error trials for channel O1, O2, and Oz; Pe = error positivity; Post.Error.Acc = post NoGo error accuracy;*

The relationships between key predictors and CBCL-Anxiety *T* scores were analyzed using partial dependence plots (PDP) (Figure 1). The most striking finding came from the Pe plot, which displayed a pronounced and consistent increase in predicted anxiety scores as Pe values increased. This steep rise suggests that an enlarged Pe during cognitive tasks is strongly associated with elevated anxiety in young children. Single/unpartnered mothers had higher mean predictive CBCL-Anxiety *T* scores than partnered mothers, indicating a consistent association between this sociodemographic risk factor and higher anxiety levels. Frontal theta power during NoGo stimuli showed an inverse relationship with anxiety, where lower theta power was associated with higher predicted anxiety scores. Post-error accuracy exhibited a complex relationship with anxiety, maintaining relatively stable predictions until approximately 0.9 accuracy, after which there was a sharp decrease in predicted anxiety. Occipital post-error alpha power showed an initially steep positive relationship with anxiety predictions, followed by a more gradual increase, indicating that higher alpha power following errors was associated with greater anxiety severity.

### Interactions Among Predictors

**Figure 2:**
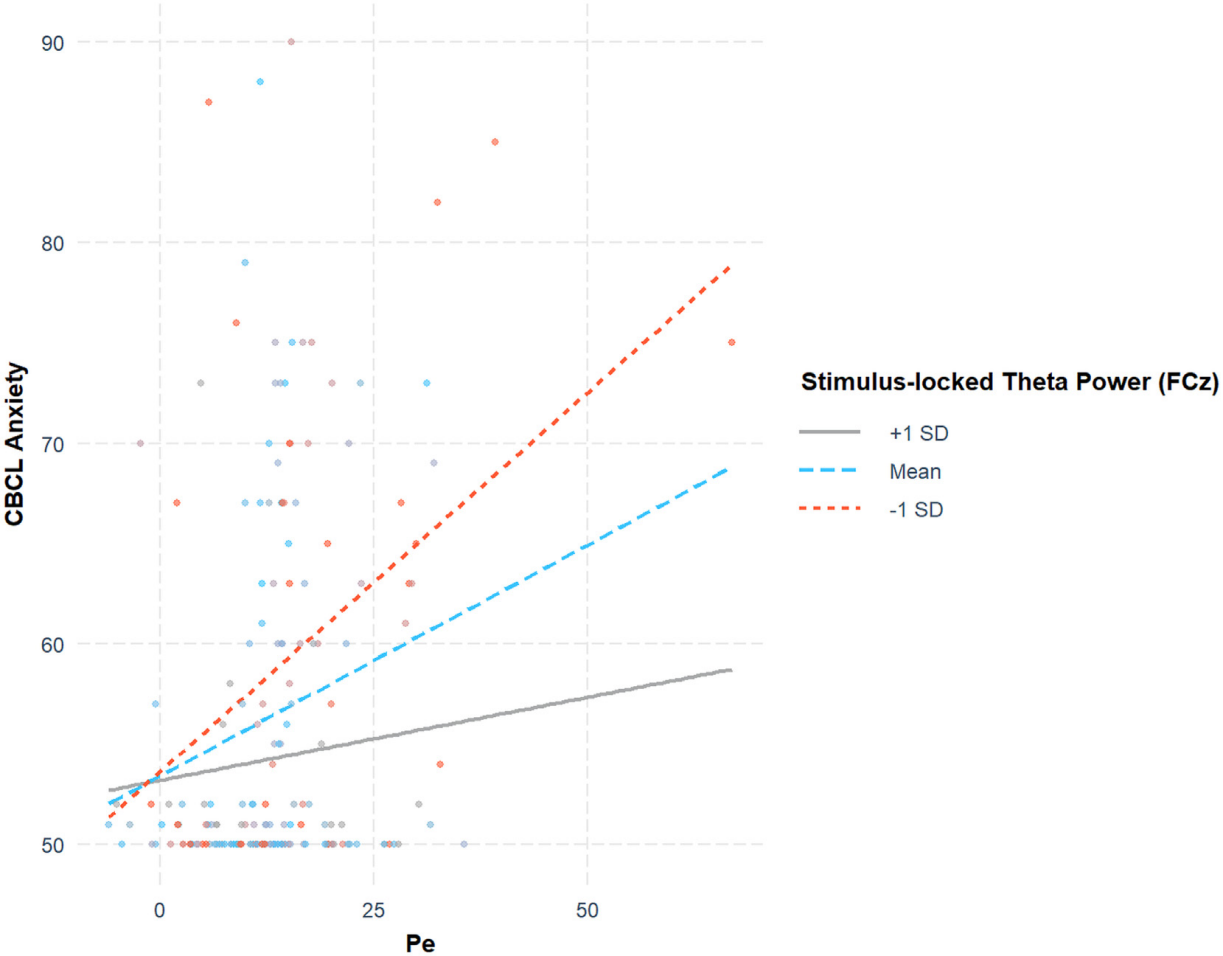
**Interaction Pe and Stimulus-Locked Theta on CBCL-Anxiety** ***Note:****Effect plot of the interaction between the predictor variable (Pe) and the moderator variable (stimulus-locked theta power for channel FCz) on the dependent variable (CBCL-Anxiety* T *scores). The plot includes observed data points and regression lines representing the effect of Pe at 3 levels of the moderator: 1 SD below the mean (−1 SD), the mean, and 1 SD above the mean (+1 SD). CBCL = Child Behavior Checklist*.

The 2-way interaction analysis using the Friedman *H* statistic revealed that the Pe interacted most robustly with the fronto-central (electrode FCz) theta power following NoGo stimulus (*H* statistic = 0.161). A moderation analysis was conducted to examine the interaction between Pe and post-NoGo stimulus-locked theta power on CBCL-Anxiety. The interaction effect between Pe and post-NoGo stimulus-locked theta power was significant (β = −0.00109, SE = 0.00047, *Z* = −2.34, *p* = .02). Simple slope analysis (Figure 2) further revealed that at low levels of post-NoGo stimulus-locked theta power (mean −1 SD), the relationship between Pe and anxiety was significant (β = 0.388, SE = 0.0886, *Z* = 4.381, *p* < .001). At the mean level of theta power, this relationship remained significant (β = 0.2319, SE = 0.0733, *Z* = 3.164, *p* = .002). However, at high levels of theta power (mean +1 SD), the relationship was not significant (β = 0.0758, SE = 0.1094, *Z* = 0.693, *p* = 0.488). These results suggest that the interaction between Pe and post-NoGo stimulus-locked theta power on anxiety is moderated by the level of theta power, with a stronger relationship at the lower end of theta power levels, but no significant relationship at higher theta power levels.

**Figure 3:**
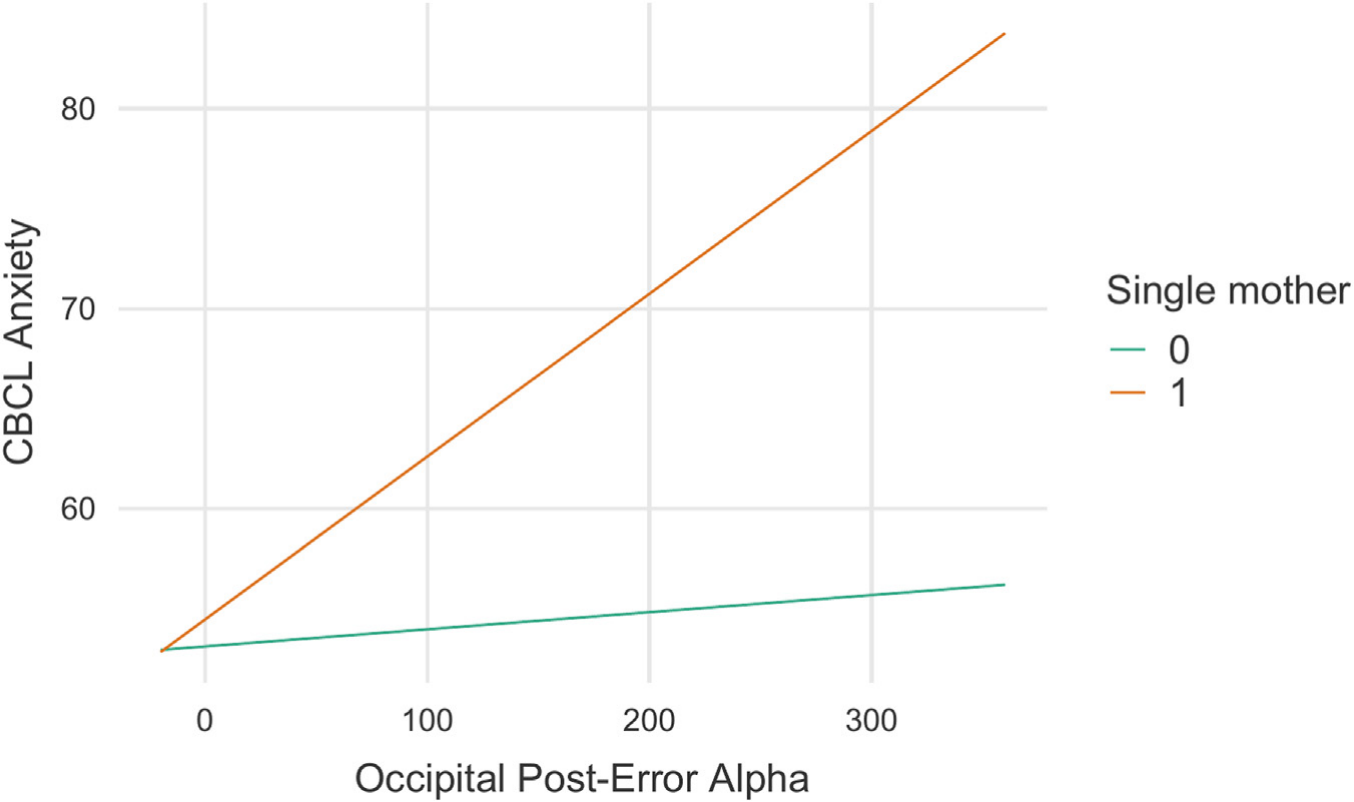
**Interaction Between Occipital Post-Error Alpha and Single-Mother Status in Predicting CBCL-Anxiety Scores** ***Note:****This plot illustrates the relationship between****occipital post-error alpha****and****CBCL-Anxiety****scores, stratified by single-parent s****tatus****(0 = partnered mother, 1 = single/unpartnered mother). A stronger positive association is observed in children of****single/unpartnered mothers****(orange line), suggesting that higher****occipital post-error alpha****after errors power (ie, less error-related alpha suppression) is more strongly linked to increased anxiety in this group compared to children of****partnered mothers****(blue line). CBCL = Child Behavior Checklist.*

The second most important predictor, single/unpartnered motherhood, showed the strongest interaction with post-error occipital alpha power (*H* statistic: 0.129). When examining the estimated marginal means in a subsequent linear model (β = 0.073, SE = 0.027, *t* = 2.67, *p* = .009), this interaction revealed that the relationship between post-error alpha power (where higher values indicate less alpha suppression following NoGo errors) and anxiety severity was substantially stronger in individuals with single/unpartnered mothers (Figure 3). Specifically, whereas individuals without this risk showed moderate increases in anxiety levels across different levels of post-error alpha power (from 53.4 at low alpha to 54.6 at high alpha), those with single mothers demonstrated a more pronounced increase in anxiety severity as post-error alpha power increased (from 56.8 at low alpha to 68.7 at high alpha), indicating reduced error-related alpha suppression. These marginal means highlight that single-mother status appears to amplify the relationship between error-related neural processing and anxiety severity.

## Discussion

The overarching goal of this study was to develop a data-driven model of childhood anxiety severity across a subclinical-to-clinical range by integrating measures across multiple levels of analysis, as outlined in the RDoC framework. Using machine learning to explore the most important predictors and interactions associated with anxiety symptoms, several key findings emerged, discussed below.

### Heightened Error Positivity (Pe) Is the Strongest Predictor of Anxiety

The Pe, an index of error awareness,^20^^,^^38^ was the strongest predictor in our model, exhibiting a consistent positive association with anxiety levels. An enlarged Pe associated with higher anxiety severity suggests a potential mechanistic link: although heightened error awareness can promote corrective behavior, in the context of anxiety, it might also intensify ruminations about mistakes. This could potentially create a cycle in which increased concern over errors exacerbates anxiety, leading to further preoccupation with mistakes, as suggested by prior research.^39^

### Lower Frontal Theta Power Reflects Poor Inhibitory Control in Anxiety

Frontal theta power following NoGo stimuli was also one of the most important predictors of anxiety severity and revealed an inverse relationship with anxiety severity. Lower theta power was associated with higher predicted anxiety scores. This finding extends previous work on cognitive control by suggesting that reduced theta oscillations may reflect compromised inhibitory control in anxiety.^40^^,^^41^ Our analysis also revealed a significant interaction between frontal theta power and the Pe in predicting anxiety levels. Specifically, low frontal theta power during NoGo stimuli interacted with enlarged Pe to predict greater anxiety severity. This suggests that deficiencies in inhibitory control, as indicated by frontal theta, are linked to heightened error awareness in association with higher anxiety symptoms. Stimulus-locked theta power during response inhibition is known to reflect the capacity for inhibitory control,^40^^,^^41^ and deficits in this process have been associated with internalizing problems such as anxiety.^6^ In theory, error awareness (indexed by the Pe) should engage mechanisms for inhibitory control (indexed by frontal theta) to prevent further mistakes.

### Reduced Post-Error Accuracy Is Associated with Higher Anxiety

Post-error accuracy also emerged as one of the top predictors of anxiety severity in our model, with less task accuracy after errors of commission predicting higher anxiety. Previous studies have shown that anxiety affects cognitive performance primarily through its effect on attentional processes.^42^ In addition, anxiety may lead to resource competition between task-related cognitive processes and anxiety-related thoughts or worries, increasing the effort needed to perform tasks correctly.^27^ Alternatively, it is possible that deficits in cognitive control, reflected in reduced post-error accuracy, may drive heightened anxiety.^6^^,^^26^ Longitudinal studies are necessary to determine whether impaired post-error accuracy is best understood as a cause or an effect of childhood anxiety. Our finding that more anxious children showed lower post-error accuracy alongside heightened Pe and reduced frontal theta suggests a possible failure in brain-based error signaling. This pattern may reflect deficient inhibitory control and impaired communication between these systems, potentially underlying mechanisms of childhood anxiety.

### Error-Related Alpha Suppression Predicts Anxiety, Amplified by Single-Parent Status

Our findings demonstrate that error-related neural processing and anxiety interact in a complex manner, particularly when considering psychosocial risk factors. We found that higher post-error alpha power (ie, reduced alpha suppression following errors) significantly predicted anxiety severity. Children of single/unpartnered mothers exhibited both stronger associations between post-error alpha power and anxiety, and higher anxiety levels overall. Post-error alpha suppression typically reflects attentional reorientation to external stimuli after errors.^43^^,^^44^ Thus, our results suggest impaired post-error attentional mechanisms in anxious individuals, especially those from single-mother households. This neural pattern aligns with established evidence that children in single-parent families show elevated risks for both anxiety and attention difficulties.^45^^,^^46^ Although our study did not directly assess maternal stress, previous research indicates that single mothers experience heightened chronic stress due to economic and caregiving burdens, which increases their children’s vulnerability to internalizing disorders.^47^ These stressors may compromise caregiving quality and emotional regulation, potentially affecting children's cognitive control mechanisms. Future research should examine whether maternal stress mediates the observed relationship between post-error alpha power and anxiety in children of single mothers.

Despite offering valuable insights, our study has some limitations. Although our sample included both community and clinical populations, capturing a broad range of anxiety severity—including subclinical and clinical levels—the majority of participants were from a community-based sample. However, with approximately 31% of children showing clinically significant anxiety symptoms, our findings may not fully capture patterns typically seen in purely clinical settings. Similarly, although all data were collected at the same site by the same team using identical protocols, differences in time of data collection across studies may have introduced unmeasured variability, which was not explicitly modeled.

In addition, our exclusive reliance on parent-reported measures of child anxiety through the CBCL presents another limitation. Although the CBCL *DSM*-oriented Anxiety Problems subscale is well validated,^31^ is commonly used, and facilitates comparison with prior work,^48^, ^49^, ^50^ future studies would benefit from incorporating multiple informants and assessment methods to provide a more comprehensive evaluation of anxiety symptoms.

Although our methodology successfully identifies general associations of electroencephalographic, behavioral, and sociodemographic variables with anxiety severity, because of its cross-sectional nature, it cannot determine whether these associations reflect cause or effect. To gain a more comprehensive understanding of how cognitive control abnormalities and other risk factors drive the evolution of early life anxiety, future research should use longitudinal designs. In addition, the use of a single cognitive task (Go/NoGo) may not have captured the full range of cognitive and affective processes relevant to anxiety. Nevertheless, by identifying established metrics of cognitive control that couple with well-known sociodemographic risk factors for childhood anxiety, our findings provide a useful foundation for future work.

A critical limitation is our sample’s underrepresentation of diverse racial and ethnic groups. Given well-documented racial and ethnic disparities in mental health care access and outcomes,^51^ future studies with more diverse samples are essential to understand how anxiety manifestation and its neural correlates may vary across different cultural and socioeconomic contexts. This expanded representation is particularly important for understanding how brain–behavioral mechanisms of cognitive control and sociodemographic disadvantage may differentially contribute to anxiety expression across diverse populations.

Future research could also benefit from incorporating additional predictors of child anxiety related to parental psychopathology. Although we included maternal depression in our model, other relevant parental factors, such as maternal stress, anxiety, and broader psychopathology, could not be analyzed because of data availability. Expanding future studies to include a more comprehensive range of factors will provide a more complete understanding of childhood anxiety development.

Finally, although our findings offer valuable knowledge on variables linked to anxiety severity in children, the associations were generally modest in this primarily community sample and are not sufficient for diagnostic use. These results should be interpreted with caution and viewed as part of the broader effort to identify multidimensional markers of childhood anxiety.

In conclusion, our analysis revealed a specific combination of neural indices of cognitive control (Pe, frontal theta, occipital post-error alpha), behavioral (post-error accuracy), and sociodemographic (single/unpartnered mother) predictors of childhood anxiety. Notably, the interaction between occipital post-error alpha and psychosocial risk, particularly single-mother status, highlighted the role of environmental factors in shaping neural vulnerability for anxiety. These findings show how cognitive control, attentional processes, and social context work together to influence childhood anxiety. Leveraging a data-driven approach allowed us to identify the most predictive and interacting factors of childhood anxiety without relying on predefined assumptions, revealing novel relationships among neural, behavioral, and environmental variables that may have been overlooked in traditional hypothesis-driven studies. Future research should use longitudinal designs, incorporate more diverse and clinically representative samples, use multi-informant assessments, and examine broader parental psychopathology to better understand the complex interplay among cognitive control deficits, sociodemographic factors, and childhood anxiety development across the full clinical spectrum. These findings suggest potential targets for early intervention by highlighting the need to consider both neural mechanisms and family context in childhood anxiety treatment strategies.

## CRediT authorship contribution statement

**Ann M. Iturra-Mena:** Writing – review & editing, Writing – original draft, Visualization, Methodology, Investigation, Formal analysis, Data curation, Conceptualization. **Melanie Wall:** Writing – review & editing, Supervision, Methodology, Investigation, Conceptualization. **Sherry Y.H. Chen:** Formal analysis. **Jason S. Moser:** Resources, Project administration, Funding acquisition, Conceptualization. **Maria Muzik:** Resources, Project administration, Funding acquisition, Conceptualization. **Katherine Rosenblum:** Resources, Project administration, Funding acquisition, Conceptualization. **Kate D. Fitzgerald:** Writing – review & editing, Supervision, Resources, Project administration, Funding acquisition, Conceptualization.
