# Supplementary material for: Data-Driven Identification of Brain–Behavioral and Sociodemographic Predictors of Anxiety Severity in Children Using Machine Learning
Source: JAACAP Open. 2025 Jun 25;3(4):1236–45. doi: 10.1016/j.jaacop.2025.05.005 (PMC12684693; doi:10.1016/j.jaacop.2025.05.005)
Supplement: Supplemental Material [file mmc1.docx]

**Supplement 1**

**The Go/NoGo Zoo Task and task performance metrics:**

In the Go/No-Go Zoo Task, ^1^ children helped a zookeeper by pressing a button to capture loose animals (Go trials) and refrained from responding to orangutans (NoGo trials). The task included eight blocks, each with 30 Go and 10 NoGo trials, for a total of 320 trials. Each trial began with a fixation cross (200–300 ms), followed by an animal image (750 ms), and then a blank screen (500 ms). Responses were allowed during the image and blank screen periods. Each block introduced a new set of animal images, balanced for size, color, and species. Before EEG data collection, children practiced with 12 trials (three orangutan and nine other animals) until they were proficient. E-prime software (Psychology Software Tools, Inc.: Pittsburgh, PA) was used for task presentation. Correct trials required pressing the button for non-orangutan animals (Go trials) and not responding to orangutans (NoGo trials). Errors included button presses during NoGo trials (false alarms, errors of commission) and non-responses during Go trials (misses, errors of omission). Post-error slowing was measured using a previously described approach. ^2^ Trials with reaction times (RT) under 100 ms were excluded to eliminate anticipatory responses. We identified specific five-trial sequences (hit, hit, false alarm, hit, hit) and calculated post-error RT from the first hit following a false alarm. Post-NoGo error accuracy was calculated by dividing the correct post-NoGo error responses by the total post-NoGo error trials. False alarm and miss rates were computed separately by dividing the total count of each error type by the total number of trials.

**Electrophysiological Recording, Data Reduction, and Analysis:**

| EEG data were recorded using the ActiveTwo system (BioSemi Inc.) with 17 Ag/AgCI scalp electrodes. BrainVision Analyzer, Version 2.2.2 (Brain Products GmbH, Gilching, Germany) was used for analysis. EEG data were sampled at 1024 Hz, band-pass filtered (0.05-30 Hz) using zero-phase shift Butterworth filters and referenced to averaged mastoid electrodes. Ocular movement artifacts were corrected with a regression-based algorithm. For post-error metrics, data were response-locked to correct Go and incorrect NoGo trials. Incorrect Go and correct NoGo trials were excluded due to the lack of a response button needed for EEG linkage in this task. Epochs were rejected if voltage steps exceeded 50 μV in 200 ms, or if trial voltage variation per channel was greater than 150 μV or less than 0.5 μV. A Current Source Density (CSD) transformation was applied to reduce the effect of volume conduction and have a “reference-free” representation of the EEG signal. ERPs were quantified using mean amplitude measurements relative to a pre-response baseline -200 to −100 ms. The mean amplitude of the ERN at channel FCZ and the Pe at channel Pz were computed 25–75 ms and 300-500 ms after NoGo errors, respectively.  The N200 and P300 data were stimulus-locked to Go and NoGo trials. For the N200, we used the mean amplitude in the 185-300 ms time window following the NoGo stimulus, and for the P300, the mean amplitude in the 250-500 ms window following the NoGo stimulus at channel FCz was used. |
| --- |

Power spectra were processed using a continuous wavelet, computed for single trials and subsequently averaged. Data from 1 to 30 Hz were transformed using a complex Morlet wavelet with a Morlet parameter c of 3.5 applied in 30 frequency steps distributed on a logarithmic scale. To apply a percentage change baseline correction of power, the corresponding stimulus or response-locked amplitude was first averaged to a pre-stimulus or response baseline on a trial-by-trial basis (-200 to −100 ms). Computed average alpha (8-14 Hz) from channels O1, O2 and Oz and theta power (4-7 Hz) from channel FCz were extracted 200–500 ms and 100-300 ms post-response, respectively. Stimulus-locked theta (4-7 Hz) at 150-400 ms power for NoGo trials was computed for channel FCz. Additionally, post-error frontal synchrony was computed in BrainVision Analyzer using the phase locking value for electrode pairs F3 and F4, F3 and FCz and F4 and FCz following NoGo errors.

**Supplement 2**

**Imputation procedure:**

Our dataset included predictors with some missing data (Table S1), while the dependent variable had no missing data. To handle missing data we utilized the missForest method. ^3^ missForest builds a random forest model for each column with missing values using all other columns as predictors. The procedure iterates multiple times to enhance accuracy, is suitable for mixed-type data, and is able to maintain data patterns while estimating missing values. To assess imputation performance, the normalized root mean squared error (NRMSE), which ranges from 0 to 1 with values closer to 0 indicating better predictive accuracy, ^4^ was used. Cut-off values for this index to indicate excellent accuracy do not exist, however typical applications have values <0.50. ^5^ We obtained a NRMSE of 0.251. We used R missForest package (version 1.5) and following standard recommendations, the parameters set for missForest were: ntree = 50, maxiter = 5, mtry = sqrt(p). ^6^

**Table S1**

|  | **Variable** | **Description** | **Data modality** | **Percent missing** |
| --- | --- | --- | --- | --- |
| 1 | **ERN** | Error-Related Negativity mean amplitude at 25 to 75 ms channel FCz | EEG - post-error ERP component | 24.86 |
| 2 | **Pe** | Error-Positivity at 300-500 ms channel Pz | EEG - post-error ERP component | 26.52 |
| 3 | **P300** | Stimulus-locked (250-500 ms) mean amplitude following NoGo errors channel FCz | EEG - post-stimulus ERP component | 34.81 |
| 4 | **N200** | Stimulus-locked (185-300 ms) mean amplitude following NoGo errors channel FCz | EEG - post-stimulus ERP component | 34.25 |
| 5 | **FCz.Post.Error.Theta** | Response-locked (100-300 ms) theta (4-7 Hz) power for NoGo trials channel FCz | EEG - post-error spectral component | 27.62 |
| 6 | **Occipital.Post.Error.Alpha** | Response-locked (200-500 ms) average alpha (8-14 Hz) power for NoGo error trials for channel O1, O2 and Oz | EEG - post-error spectral component | 27.62 |
| 7 | **FCz.Theta.Stim.NoGo** | Stimulus-locked (150-400 ms) theta (4-7 Hz) power for NoGo trials channel FCz | EEG - post-stimulus spectral component | 28.73 |
| 8 | **PLV_F3.F4_Alpha_NoGo_Error** | Post-error (0-200 ms) alpha synchrony: Phase locking value (PLV) for electrode pair F3 and F4 following NoGo errors. | EEG - post-error synchrony measure | 37.02 |
| 9 | **PLV_F3.FCz_Alpha_NoGo_Error** | Post-error (0-200 ms) alpha synchrony: PLV for electrode pair F3 and FCz following NoGo errors. | EEG - post-error synchrony measure | 37.02 |
| 10 | **PLV_F4.FCz_Alpha_NoGo_Error** | Post-error (0-200 ms) alpha synchrony: PLV for electrode pair F4 and FCz following NoGo errors. | EEG - post-error synchrony measure | 37.02 |
| 11 | **PLV_F3.F4_Theta_NoGo_Error** | Post-error theta synchrony: PLV for electrode pair F3 and F4 following 25-75 ms of NoGo errors. | EEG - post-error synchrony measure | 37.02 |
| 12 | **PLV_F3.FCz_Theta_NoGo_Error** | Post-error theta synchrony: PLV for electrode pair F3 and FCz following 25-75 ms of NoGo errors. | EEG - post-error synchrony measure | 37.02 |
| 13 | **PLV_F4.FCz_Theta_NoGo_Error** | Post-error theta synchrony: PLV for electrode pair F4 and FCz following 25-75 ms of NoGo errors. | EEG - post-error synchrony measure | 37.02 |
| 14 | **Post.Error.RT** | Post NoGo error reaction time in the Zoo Task | Performance Go/NoGo task | 27.62 |
| 15 | **Post.Error.Acc** | Post NoGo error accuracy | Performance Go/NoGo task | 27.62 |
| 16 | **miss.ER** | Zoo Task misses (error of omission) error rate | Performance Go/NoGo task | 16.02 |
| 17 | **fa.ER** | Zoo Task false alarm (error of commission) error rate | Performance Go/NoGo task | 16.02 |
| 18 | **hit.RT** | Zoo Task reaction time, correct go trials | Performance Go/NoGo task | 16.02 |
| 19 | **fa.RT** | Zoo Task reaction time errors of commission | Performance Go/NoGo task | 16.02 |
| 20 | **BDI.score** | Beck Depression Inventory (BDI) sum of all items | Self-report mother | 0.55 |
| 21 | **Sex** | Sex at birth | Sociodemographic | 0 |
| 22 | **partneredrisk** | Indicator of sociodemographic risk: single or unpartnered mother (1 = risk, 0 = no risk). | Sociodemographic | 0 |
| 23 | **edurisk** | Indicator of sociodemographic risk: low maternal education (1 = risk, 0 = no risk). | Sociodemographic | 0 |
| 24 | **incomerisk** | Indicator of sociodemographic risk: low household income, less than $25,000 (1 = risk, 0 = no risk). | Sociodemographic | 0 |
| 25 | **racerisk** | Indicator of sociodemographic risk: racial minority status (1 = risk, 0 = no risk). | Sociodemographic | 0 |
| 26 | **Age** | Age in months | Sociodemographic | 0 |
| DV | **CBCL-Anxiety** | Child Behavior Checklist (CBCL) DSM Oriented Scale T-scores. | Parent report on child | 0 |

**Table S1:** Variables included in the Random Forest model and missing data. 26 predictors were included in the random forest model. The dependent variable was the Child Behavior Checklist (CBCL) DSM Oriented Scale Anxiety T-scores. Missing data is presented as percentage.

**Table S2**

| **Feature** | **mean (SD)** |
| --- | --- |
| miss.ER | 0.04 (0.04) |
| fa.ER | 0.08 (0.04) |
| hit.RT | 581.83 (77.13) |
| fa.RT | 470.50 (69.38) |
| Post.Error.RT | 526.89 (57.04) |
| Post.Error.Acc | 0.89 (0.06) |
| Pe | 13.84 (9.13) |
| ERN | -3.08 (5.31) |
| N200 | -11.19 (7.18) |
| P300 | -14.18 (8.75) |
| FCz.Theta.Stim.NoGo | 311.45 (143.84) |
| Occipital.Post.Error.Alpha | 102.30 (62.99) |
| FCz.Post.Error.Theta | 137.46 (85.58) |
| PLV_F3.F4_Alpha_NoGo_Error | 0.32 (0.11) |
| PLV_F3.FCz_Alpha_NoGo_Error | 0.30 (0.10) |
| PLV_F4.FCz_Alpha_NoGo_Error | 0.29 (0.12) |
| PLV_F3.F4_Theta_NoGo_Error | 0.37 (0.12) |
| PLV_F3.FCz_Theta_NoGo_Error | 0.30 (0.12) |
| PLV_F4.FCz_Theta_NoGo_Error | 0.29 (0.12) |

**Table S2:** **Neural Features and Zoo Task Performance**. Summary of Neural Features and Zoo Task Performance, highlighting mean values and standard deviations for the neural and task-related metrics included in the machine learning models.

**Table S3**

| Risk Factor | N | White non-Hispanic or Latino | Black or African American | Hispanic or Latino | Native American | Asian or other Pacific Islander | Bi-Racial | Other | Test Statistic |
| --- | --- | --- | --- | --- | --- | --- | --- | --- | --- |
|  |  | **(N=124)** | **(N=20)** | **(N=5)** | **N=0** | **(N=5)** | **(N=26)** | **(N=1)** |  |
| Single mother | 181 | 0.3   38/124 | 0.6  11/20 | 0.2  1/5 | 0.0  0/0 | 0.4  2/5 | 0.3   8/26 | 0.0  0/1 | Χ25=5.72, P=0.33^2^ |
| Low education | 181 | 0.0   4/124 | 0.0   0/20 | 0.0  0/5 | 0.0  0/0 | 0.2  1/5 | 0.0   0/26 | 0.0  0/1 | Χ25=7.11, P=0.21^2^ |
| Low income | 181 | 0.0   5/124 | 0.3   6/20 | 0.0  0/5 | 0.0  0/0 | 0.0  0/5 | 0.2   5/26 | 1.0  1/1 | Χ25=27.81, P<0.01^2^ |

**Table S3.** **Distribution of sociodemographic risk factors by racial/ethnic groups.** The table presents the proportion of participants from each racial/ethnic category who fall into key risk factor groups, including single motherhood, low maternal education, and low income. Chi-square tests were conducted to assess group differences, with significant differences observed for low income (Χ²₅ = 27.81, P < 0.012), while single motherhood and low maternal education did not show significant variation across racial/ethnic groups.

**Table S4:**

| **Model** | **Mean R2** | **CI Lower** | **CI Upper** |
| --- | --- | --- | --- |
| RF | 0.926 | 0.900 | 0.947 |
| SVR | 0.632 | 0.502 | 0.757 |
| XGB | 0.620 | 0.529 | 0.705 |
|  | | | |
| **Comparison** | **Mean Diff** | **P Value** | **Significant** |
| RF vs SVR | 0.294 | < .001 | Yes |
| RF vs XGB | 0.307 | < .001 | Yes |
| SVR vs XGB | 0.013 | 0.888 | No |

**Table S4:** **Bootstrap Comparison of Machine Learning Model Performance.** The table shows results from bootstrap resampling analysis (1000 iterations) comparing the performance of Random Forest (RF), Support Vector Regression (SVR), and XGBoost (XGB) models for predicting CBCL-Anxiety T-scores. The top section shows mean R² values with 95% confidence intervals for each model. The bottom section displays pairwise statistical comparisons between models, including mean R² differences, p-values, and significance determinations (α = 0.05). Bootstrap resampling involved repeatedly drawing samples with replacement from the original dataset, running each model on these samples, and calculating performance metrics to generate empirical distributions for statistical comparison.

**References:**

1. Grammer JK, Carrasco M, Gehring WJ, Morrison FJ. Age-related changes in error processing in young children: a school-based investigation. *Dev Cogn Neurosci*. Jul 2014;9:93-105. doi:10.1016/j.dcn.2014.02.001

2. Rueppel M, Mannella KA, Fitzgerald KD, Schroder HS. Post-error slowing in anxiety and obsessive-compulsive disorders. *Cognitive, Affective, & Behavioral Neuroscience*. 2022:1-15.

3. Stekhoven DJ, Bühlmann P. MissForest—non-parametric missing value imputation for mixed-type data. *Bioinformatics*. 2012;28(1):112-118.

4. Oba S, Sato M-a, Takemasa I, Monden M, Matsubara K-i, Ishii S. A Bayesian missing value estimation method for gene expression profile data. *Bioinformatics*. 2003;19(16):2088-2096.

5. Kokla M, Virtanen J, Kolehmainen M, Paananen J, Hanhineva K. Random forest-based imputation outperforms other methods for imputing LC-MS metabolomics data: a comparative study. *BMC bioinformatics*. 2019;20:1-11.

6. Boehmke B, Greenwell BM. *Hands-on machine learning with R*. Chapman and Hall/CRC; 2019.
